# Enteral Nutrition in Neonatal Cholestasis: An Up-to-Date Overview

**DOI:** 10.3390/nu17111794

**Published:** 2025-05-26

**Authors:** Elisa Cimadamore, Martina Palazzo, Maria Chiara Fioroni, Martina Cerverizzo, Alessio Correani, Ilaria Burattini, Chiara Biagetti

**Affiliations:** 1 Department of Odontostomatologic and Specialized Clinical Sciences, Università Politecnica delle Marche, 60123 Ancona, Italy; elisa.cimadamore@pm.univpm.it (E.C.); mariachiarafioroni@gmail.com (M.C.F.); cerverizzomartina@gmail.com (M.C.); a.correani@staff.univpm.it (A.C.); 2 Division of Neonatology, Mother and Child Department, G. Salesi Children’s Hospital, Azienda Ospedaliero-Universitaria delle Marche, 60123 Ancona, Italy; martinapalazzo18@gmail.com (M.P.); ilaria.burattini@ospedaliriuniti.marche.it (I.B.)

**Keywords:** intestinal failure, cholestasis, chronic liver disease, enteral nutrition, nutritional support, newborn, preterm

## Abstract

Cholestasis is an uncommon but potentially life-threatening clinical condition in the neonatal period, leading to maldigestion/malabsorption of fats and fat-soluble components of the diet. Thus, nutritional management is crucial for the cholestatic newborn in order to sustain growth and development. Even if it can be recognized a wide variety of diseases underlying neonatal cholestasis, from a nutritional point of view, patients can be categorized into two main groups, according to their intestinal integrity in length and function, which influences the nutritional strategies to be used: patients with intestinal failure-associated liver disease (IFALD) and those suffering from liver dysfunction without intestinal impairment (NOT IFALD). For both groups, enteral nutrition is widely considered a cornerstone of their care. In this narrative review, we summarize the evidence that guides neonatologists in the complex management of enteral nutrition in a cholestatic newborn, such as the choice of type of milk to be used or of any supplementation needed, focusing on preventive and curative strategies including their effects on sustaining growth. Analyzing data published over a period of more than 50 years, despite the agreement of experts and societies in many aspects of management of both IFALD and NOT IFALD cholestatic newborns, we found that robust evidence behind clinical practice is still lacking. This underscores the urgent need for well-designed multicenter randomized controlled trials to optimize the nutritional care of this vulnerable patient population.

## 1. Introduction

Cholestatic jaundice is an uncommon but potentially serious clinical problem in the neonatal period, indicating a hepatobiliary dysfunction, and deserves prompt recognition and accurate clinical and nutritional management. It occurs approximately in 1 every 2500 newborns. Among NICU admissions, up to 2% of newborns develop cholestasis, with a prevalence of about 20% in preterm infants, which are particularly at risk, due to several risk factors [1,2,3].

Cholestasis is characterized by reduced bile flow with accumulation of bile products, such as bilirubin, bile acids, and cholesterol in the liver, blood, and other tissues. Several biochemical definitions based on serum direct bilirubin concentration have been proposed in the last decades, but there is still no consensus on the cut-off value used for the definition of neonatal cholestasis in both term and preterm infant studies. The American Academy of Pediatrics (AAP) recently updated the Clinical Practice Guidelines for the Management of Hyperbilirubinemia in the newborn infant ≥ 35 weeks of gestation, incorporating the joint recommendation from the North American and European Societies for Pediatric Gastroenterology, Hepatology, and Nutrition, which defines a direct serum bilirubin concentration > 1.0 mg/dL (17.1 μmol/L) as abnormal; moreover, they state that the previous additional definition of a direct bilirubin concentration of >20% of the total is no longer regarded as necessary for the diagnosis of cholestasis [4,5]. In clinical practice, direct bilirubin serum concentrations greater than 1.0 mg/dL have been demonstrated as sufficiently accurate to indicate neonatal cholestasis [6,7,8]. However, most of the studies dealing with term and preterm infants considered a direct bilirubin concentration above 2 mg/dL as the cut-off value to define cholestasis [3,9,10,11].

The etiology of neonatal cholestasis includes a wide spectrum of clinical conditions that can be classified in nine groups, according to the main mechanism of hepatic dysfunction (Table 1) [1].

The impaired bile flow and the subsequent reduction of bile acids in the intestinal lumen are responsible for the maldigestion/malabsorption of fats and fat-soluble components of the diet, leading to steatorrhea and malnutrition [12]. In fact, in many congenital or acquired conditions associated with neonatal cholestasis, malabsorption and/or malnutrition are the predominant clinical features. Thus, nutritional management is crucial for the cholestatic infant, in order to tackle the potential detrimental effects of cholestasis on growth and development.

From a nutritional perspective, patients who suffer from cholestasis can be divided into two main groups, according to the patients’ intestinal anatomic and functional integrity, that influence the management of their nutritional support and the modalities to deliver nutrients in order to achieve body growth and adequate nutritional status.

The first group consists of patients affected by intestinal failure (IF), defined as the “reduction of functional gut mass below the necessary amount to provide adequate nutrient and fluid requirements to sustain normal body growth” [13]. Neonatal IF is mostly due to gastro-intestinal diseases, such as necrotizing enterocolitis (NEC), midgut volvulus, intestinal atresia or gastroschisis, and thrombosis, that require intestinal surgery, which often ends up in an intestinal resection of variable length and, sometimes, in the creation of an enterostomy; the subsequent clinical condition characterized by a reduction in length of the intestinal mass has been named short bowel syndrome (SBS). Other rare causes of neonatal IF are neuromuscular disorders affecting almost all the small bowel, or congenital enterocyte disorders [14]. The associated cholestatic liver disease, or intestinal failure associated liver disease (IFALD), has been defined as the “hepatobiliary dysfunction as a consequence of medical and surgical management strategies for IF” [15], and it has been connected to multiple risk factors, both related to the patients and their parenteral nutrition (PN) need [16]. In fact, IFALD patients mostly rely on PN support to ensure the availability of nutrients for growth and survival; nonetheless, the provision of enteral nutrition (EN) in these patients, even if often challenging, is a key factor to mitigate the hepatic disease associated with IF.

The second group (NOT IFALD) consists of patients who develop cholestasis because of a wide range of medical conditions which cause the impairment of liver function. Nutritional management for these patients, whose intestinal integrity is preserved, is essentially based on optimization of EN, trying to overcome the maldigestion/malabsorption related to cholestasis, in addition to the treatment of the underlying medical condition.

Whatever the categorization, preterm infants are the most vulnerable patients, because of several risk factors, like in utero growth restriction, low birth weight, intestinal immaturity or ischemia, liver immaturity with high susceptibility to insults and oxidative stress, long duration of PN to support the growth of the tiniest, potential feeding intolerance causing hampered EN and gallbladder stimulation, NEC (often requiring intestinal resections or ileostomies), high incidence of sepsis, and use of hepatotoxic medications [13]. Thus, the nutritional management of a cholestatic preterm infant can be a real challenge for neonatologists and nutritionists. Nevertheless, ensuring the most adequate nutrition to a cholestatic preterm patient is fundamental, as cholestasis is associated with growth and neurodevelopment impairment, in addition to bone disease, and can therefore have severe long-term consequences in these fragile patients [17,18].

The aim of this review is to summarize the current knowledge available about the EN support in newborns with IFALD or NOT IFALD cholestasis, analyzing the evidence that still guides the neonatologists’ clinical decisions. More in detail, this review will focus on three different aspects of the relationship between EN and neonatal cholestasis:

- The role of EN on the prevention or reversal of neonatal cholestasis in IFALD cholestatic patients;
- The role of EN on the prevention of neonatal cholestasis (whenever possible) and management of NOT IFALD cholestatic patients;
- The effect of nutritional management and specific nutritional interventions on growth of both IFALD and NOT IFALD cholestatic patients.

## 2. Materials and Methods

Studies evaluating the impact of EN on cholestasis in IFALD patients, nutritional strategies in NOT IFALD patients and the effect of EN on their growth were considered eligible. Inclusion criteria were as follows: published studies (with no publication date imposed) involving term and preterm infants (0–28th day after the completion of 40 weeks post-menstrual age) affected by IFALD or NOT IFALD cholestasis. Cross-sectional, cohort, case–control, case reports, and series were included. Abstracts presented at meetings and articles not published in English were excluded. Studies in animal models or in infants and children were evaluated and included if judged relevant to the review. An extensive literature search was performed in PubMed until March 2025.

The following keywords and search terms were used: cholestasis, parenteral nutrition-associated cholestasis, parenteral nutrition-associated liver disease, intestinal failure associated liver disease, intestinal failure OR small bowel syndrome, chronic liver diseases AND enteral nutrition, nutritional management, nutritional strategies, enteral approaches, malabsorption, intestinal rehabilitation, postoperative feeding, AND newborn, preterm infant, infant. In addition, references of the selected studies were screened to identify other relevant articles.

## 3. The IFALD Cholestatic Patient

### 3.1. Definition

Cholestasis in a neonatal intensive care unit can be part of a well-defined clinical entity, where a patient develops a liver disease as a consequence of IF (IFALD). This clinical scenario is different from the self-recovering cholestasis that affects up to 30% of preterm patients, which is related to short-term PN and extreme prematurity, usually named parenteral nutrition-associated liver disease/cholestasis (PNALD/PNAC) [10]. However, the three terms, IFALD and PNALD/PNAC, have been mutually used in previous decades, thus leading to heterogenicity in the available literature and possibly to misunderstandings.

Despite having introduced the term IFALD, there is no established definition of liver disease in this setting and it is unclear whether IFALD should be diagnosed based on clinical, biological, or histological criteria. Lately, most of the studies defined IFALD as a plasma direct bilirubin concentration above 2 mg/dL for at least two consecutive measurements in patients receiving PN for at least 14 days. Actually, these studies included infants born preterm on PN for months. However, there are also studies in which patients on PN for 7 days were considered eligible, based on the authors’ observation of early cholestasis development in some infants [19].

Some studies tried to categorize the early stages of the disease, and a plasma direct bilirubin concentration above 1.2 mg/dL has been considered an initial sign of liver disease; other authors have defined the presence of transaminases and/or gamma-glutamyl-transferase (GGT) (>1.5 upper limit of normal) associated with an hyperbilirubinemia below 3 mg/dL or even a mild isolated raise in transaminases and a moderate hyper-GGT (≤4 upper limit of normal) as early IFALD [13].

### 3.2. Incidence

The absence of a clear definition in association with heterogenicity of the published literature makes it difficult to estimate the exact prevalence of IFALD in the neonatal and pediatric population. A systematic review by Lauriti et al. that identified and separately considered papers on PNAC and IFALD in the available literature found an incidence of IFALD of 49.8% in pediatric patients; among the studies included in the review, only one paper published in 2005 reported data exclusively on newborns, finding an IFALD incidence of 62% [10]. Over the last 20 years, more recent studies have shown a prevalence of 20% in children on home PN [15,20], but data available on neonates are still scarce. Of note, it should be considered that studies on neonates are mainly on preterm infants, in whom liver immaturity, the 24 h continuous infusion of PN, and a high incidence of sepsis and NEC facilitate liver inflammation and severe damages, likely contributing to the higher incidence of IFALD described in newborns [15].

### 3.3. Etiology

IF can be caused by several disorders of the gastrointestinal tract, which have been categorized into three main groups: anatomical (i.e., SBS as a consequence of NEC, midgut volvulus, intestinal atresia or gastroschisis, and thrombosis), neuromuscular (i.e., Hirschsprung disease/total aganglionosis or other neuromuscular motility disorders extensively affecting the small bowel) or mucosal intestinal diseases (i.e., congenital diarrhea and other congenital enteropathies) [21] Congenital enterocyte disorders and small bowel neuromuscular diseases are mostly associated with established or progressive irreversible IF in addition to liver disease, thus making it difficult to advance the enteral feeding; therefore, these conditions often require intestinal and/or liver transplantation. On the contrary, extensive intestinal resections and SBS, which account for the majority of neonatal IFALD cases [22], can more often be managed with an intestinal rehabilitation program to achieve enteral autonomy; therefore, even if it is still a potential cause of end-stage liver disease (ESLD), IFALD in SBS can be a preventable or reversible liver disorder.

### 3.4. The Role of Enteral Nutrition in the Prevention or Reversal of IFALD

EN plays a key role in the management of “IFALD cholestatic patients”, and it is considered an important tool for the prevention or reversal of IFALD. In fact, it represents the cornerstone of the intestinal rehabilitation in newborns with intestinal resections and SBS, as well as for patients with enterostomy. It promotes the adaptation of the remnant intestine, stimulating structural and functional changes of the intestinal mucosa; in addition, the provision of nutrients in the intestinal lumen enhances the enteral blood flow, and promotes the release of gastric, pancreatic, and biliary secretions, intestinal hormones, and neural factors [23]. Hence, there is general consensus that the transition from PN to EN should be done as soon as possible and supported to its maximum extent to reduce the PN burden, which is a major contributor to cholestatic liver disease in these patients. However, EN management and the success of the transition are highly influenced by multiple factors, that should be carefully evaluated in each single patient: the length and functional integrity of the remnant intestine, the site of the resection, the presence or absence of the ileo-cecal valve and/or the colon, the creation of an enterostomy. Therefore, due to the wide range of clinical conditions in these patients, controversy exists on the best feeding strategy, regarding the type of milk, mode of delivery, initiation of feeding and feeding advancements, and nutrients requirements.

**Figure 1:**
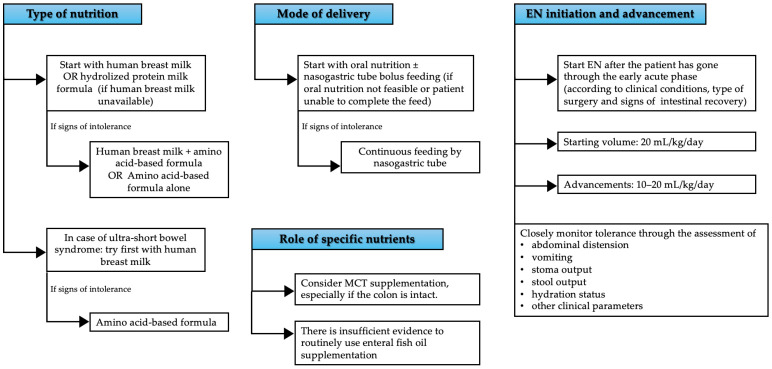
A practical approach to feeding IFALD newborns.

In fact, despite the widespread awareness of its importance in patients affected by IF and even more in cholestatic patients, there is little data on dietary management of IFALD cholestatic newborns; the available literature is mostly retrospective in nature and focused on patients with SBS, trying to find associations between several factors, including aspects of EN management, and the duration of PN. Most studies focus on preterm born infants, as one of the leading causes of intestinal failure is NEC, that has been retrospectively studied after varying durations of PN support, ranging from 14 to 90 days, and even longer in some case reports. Data on liver disease in these patients are often lacking or incomplete. In addition, we found no studies investigating dietary management in neonates with ileostomies, which are often—but not consistently—included among study patients in the available literature. Therefore, the real impact of EN on the prevention or reversal of cholestatic liver disease remains difficult to ascertain. The evidence we found on the impact of EN on the prevention or treatment of IFALD is described in the following sections and summarized in Table 2; a practical approach to feeding IFALD newborns is suggested in Figure 1.

#### 3.4.1. Human Milk Versus Infant Milk Formulas

Data about the type of milk to be chosen to prevent or treat IFALD, even if the topic has been of interest during the last 50 years, are still scarce and of a low level of evidence.

Only one retrospective study investigated the association between the type of EN and the incidence of PNALD in a neonatal intensive care unit. The authors found that the prevalence of PNALD was significantly lower in infants who were fed only human breast milk (34.6%) compared with those who were fed only milk formula (72.7%; *p* = 0.008). Moreover, the mean maximum plasma conjugated bilirubin concentration was significantly lower in the breastfed infants compared to the formula fed group, suggesting that human breast milk was protective against the development of PNALD in infants receiving PN for >4 weeks [25].

Another study reported that enteral feeding with human breast milk had the strongest correlation in the univariate analysis, with a shorter duration of PN in neonates with SBS; however, after adjusting for multiple confounders, only the number of days without intestinal continuity remained significantly associated with PN duration. Notably, human breast milk was not associated with the peak concentration of direct bilirubin [26].

Even if evidence about the potential role of human milk in the prevention or treatment of IFALD is still of very low-grade, there is general consensus that the use of human breast milk should be the first choice for patients with IF/SBS, with or without associated liver disease, mainly because of the well-established knowledge about human milk benefits for the mother–child dyad [35,36]. Actually, the positive effects of human breast milk on intestinal rehabilitation are thought to be related to the presence of non-nutritive factors, such as immunoglobulin A, leucocytes and nucleotides, anti-inflammatory cytokines, growth-factors, human milk oligosaccharides, and microbiota [35]. Moreover, some studies have suggested the beneficial effect of human breast milk, which contains glutamine and growth factors, on intestinal adaptation and epithelial cellular growth [37,38].

Given the importance of human breast milk, the potential role of pasteurized donor human milk has been widely studied in newborn nutrition over the past decades, especially for the very low birth weight infant. However, no studies exploring the use of pasteurized donor human milk in patients with IFALD were found.

If human breast milk is not available, an infant milk formula should be started. Which formula is the best option for these patients is still a matter of debate. We could not find any randomized clinical trial (RCT) dealing with this issue in IFALD cholestatic newborns. Almost 50 years ago, Adibi et al. showed that dipeptides were better absorbed than single amino acids when administered by jejunal perfusion in healthy human volunteers [39]. On these bases, hydrolyzed protein formulas have been popularized for use in patients in whom absorptive capacity is limited. However, scant data are available about the use of hydrolyzed or amino acid-based formulas in relation to their impact on IFALD in newborns. In a retrospective study conducted in 2001 in preterm born infants on PN from at least 90 days, the authors found that enteral feeding with a protein hydrolysate was associated with a lower peak direct bilirubin concentration; moreover, the amino acid-based formula was significantly associated with reduced PN duration in the univariate analysis. However, these associations were not confirmed in multivariate analyses [26]. In a retrospective study, including four patients (newborns and infants) with SBS, the use of an amino acid-based formula was supposed to have induced an earlier weaning from PN, but no mention was made on the liver status of these patients [40]. The same conclusion was also drawn in a previous case series in children on long-term PN [41]. However, the very small sample sizes and the lack of control groups in these studies do not support the recommendation of using amino acid-based formulas in SBS patients. Moreover, we found only one prospective, randomized, cross-over, double blind study in ten patients (age: 6 weeks to 8 months; cholestasis: 3 out of 10) with SBS, demonstrating neither absorptive advantages nor differences in energy expenditure or weight gain in administering hydrolyzed vs. non-hydrolyzed proteins; no other clinical outcomes or laboratory findings were evaluated in these patients [24]. Interestingly, this finding has been supported by another more recent study that showed an intact intestinal protein absorptive capacity in neonates with an enterostomy after bowel resection [42]. Finally, in a review by Capriati et al. including 10 studies and 822 infants on PN from at least 3 months, the authors found that the duration of PN was not influenced by the initial diet of the patients (hydrolyzed formula vs. human milk + hydrolyzed formula vs. human milk + amino acid-based formula) [43].

Overall, evidence on the use of infant milk formulas and their effect on IFALD is still lacking. Therefore, recommendations are often more experience-based than evidence-based. When human breast milk is not available, many clinicians choose to feed their patients with hydrolyzed or amino acid-based formulas, the latter being preferred in case of failure of the hydrolyzed formula to wean the patient off PN or when they are dealing with an ultra-short bowel syndrome [44,45].

#### 3.4.2. Mode of Delivery: Oral Versus Tube Feeding

In neonates with IF, feeding strategies aiming at achieving enteral autonomy and preventing or treating complications from PN, such as IFALD, vary widely across centers due to a lack of clear guidelines [46]. There is general agreement that, whenever feasible, EN should be administered orally to promote swallowing motor skills and help prevent feeding aversion behaviors [47,48]. Moreover, there is some evidence that suggests a potential preventive role of oral feeding on IFALD. In fact, it has been shown in animal models of SBS that oral feeding stimulates the release of vascular and epidermal growth factors (VEGF, EGF) from the salivary glands and enhances the production of gastrointestinal trophic factors. These effects accelerate intestinal adaptation by improving vascular density and the villus–vessel area ratio [49,50]. When oral feeding is unfeasible (e.g., due to mechanical ventilation, immaturity, hemodynamic instability, intestinal dysmotility), early oral stimulation is essential to prevent feeding disorders, even though nutritional support must be provided via a feeding tube. In such case, nasogastric or gastrostomy tube feeding is preferred, as these methods allow bolus feeding and support oral motor training, facilitating the transition to oral nutrition [48]. Conversely, jejunal feeding should be reserved for neonates with gastric dysmotility, severe gastroesophageal reflux, and a high risk of aspiration, given the higher risk of diarrhea and intussusception associated with J-tubes, as well as their inability to support bolus feeding [51,52,53,54].

#### 3.4.3. Mode of Delivery: Bolus Versus Continuous Feeding

Although oral feeding is widely considered a favorable approach when it comes to deciding the optimal strategy for delivering EN in patients with IF, and even if it is judged feasible in the specific circumstance, there still might be doubts for clinicians on whether a tube feeding regimen may be a better choice for these patients. In fact, it has been hypothesized that prolonged mucosal contact with nutrients enhances absorption by maximizing protein carrier saturation. Studies in pediatric patients with IF have demonstrated that continuous enteral feeding is more effective than intermittent feeding in optimizing the absorption of lipids, proteins, and energy, as well as in promoting intestinal adaptation [55,56,57]. However, the absence of fasting periods may compromise intestinal motility, gallbladder emptying, neuromuscular function, and gastric acid secretion, potentially increasing the risk of small intestinal bacterial overgrowth (SIBO) and IFALD [51,58]. Moreover, intermittent feeding is more physiological, inducing cyclical variations in plasma levels of gastrointestinal hormones (such as insulin, pancreatic polypeptide, GIP, gastrin, motilin, enteroglucagon, and neurotensin), which promote intestinal adaptation, motility, regular gallbladder emptying, and oro-motor skill development when administered orally [48,59,60]. In light of these pathophysiological considerations, and in the absence of controlled studies in newborns and children, practices vary considerably. A recent survey by the ERNICA consortium revealed that fewer than half of the surveyed centers use intermittent feeding, while the majority employ continuous or combined regimens [46].

This approach, indeed, is in line with experts and pediatric nutrition societies recommendations, which suggest using a combined approach with continuous EN overnight and bolus feeding during the day. This regimen has proven feasible and may help shorten the duration of PN, accelerate enteral autonomy, and consequently reduce the incidence of IFALD [15,35,48,51,61,62,63].

#### 3.4.4. Initiation of Feeding

In the late 80s, two RCTs that enrolled very low birth weight infants showed evidence of a protective role of early enteral feeding on PN-associated liver damage [64,65]. After these findings, a study based on an animal model confirmed that enteral supplementation was protective against liver damage induced by PN and demonstrated that alterations of gut hormones are involved in its pathogenesis [66]. Despite this first evidence, prospective RCTs on when to start enteral feeding and the speed of increment of enteral feeds in IF or IFALD patients are not currently available. In the last two decades, retrospective studies have shown that early enteral feeding improves postoperative outcomes in newborns by enhancing feeding tolerance, reducing the time to first bowel movements, and decreasing both the duration of PN and the incidence of cholestasis [27,32,48,67]. Moreover, several cohort studies demonstrated a correlation between prolonged enteral fasting and an increased risk of IFALD in both surgical and non-surgical neonates [32,68]. Specifically, in a cohort of 91 preterm infants with IF secondary to surgical NEC (Bell stage III), the time from surgery to enteral feeding initiation and the duration of postoperative ileus were independently associated with direct bilirubin levels between 2 and 5 mg/dL (mild to moderate cholestasis) at two months of age. Infants who developed cholestasis had a mean time to resume enteral feeding of 21 days, compared to 14 days in those without cholestasis [32].

Early feeding has been defined as initiation of enteral feeding within 48 h [48,69]. Of interest, we found only one RCT where surgical neonates received early enteral feeding (3 to 5 mL of human breast milk every hour through a nasogastric tube) initiated at a mean of 12 h post-surgery regardless of bowel movement; in this study, the authors found that patients in the intervention group achieved earlier stool passage, and in the subgroup of neonates with surgical anastomosis also shorter nasogastric feeding duration, faster full oral feeding and reduced hospital stay were observed in comparison with those whose feeding was delayed until ileus resolution. Even if no significant differences in plasma total bilirubin levels were observed, these findings suggest the potential role of early enteral feeding in reducing PN dependence and preventing IFALD [27]. Based on the available evidence, the ESPGHAN position paper on IFALD recommends that EN should be initiated as early as possible in addition to PN, when the neonate is stable (usually when he has gone through the early acute phase), and advanced to the maximum tolerated volume, with early feeding generally defined as starting within 48 h after surgery [15,69]. However, the ERNICA survey showed that only 25% of centers begin EN within 24–48 h post-surgery, while others wait for the first stool passage or rely on clinical judgment [46]. In fact, the optimal timing for enteral feeding initiation remains debated. Some studies recommend starting with the return of peristalsis, while others wait for stool passage [19,46,51,63]; conversely, other authors support initiating feeding within 24–48 h post-surgery regardless of overt signs of intestinal recovery [67].

Actually, infants with IF are a heterogeneous group of patients with different gestational ages, weight, and underlying diagnoses, varying degrees of residual bowel length and function, and possible ostomy creation during surgery. In addition, there are still concerns on when and how to refeed a patient after surgical NEC, as there are no RCTs dealing with these issues [70]. These considerations can explain the wide range of days at start of enteral feeding after surgery found in the available literature. Regarding the initial feeding volume, based on the assumption that earlier and higher initial volumes of minimal EN up to 20 mL/kg/day have been demonstrated safe in other high-risk neonatal populations, including low birth weight and preterm infants, some authors tried to analyze the impact of the implementation of standardized post-operative feeding guidelines on the incidence of IFALD in their NICU [19,28,71]. Shakeel et al. demonstrated that higher initial volumes of minimal EN, up to 20 mL/kg/day, and faster daily feeding advancements in surgical neonates requiring PN, led to faster achievement of 50% of the goal EN calories and a decrease in moderate (direct bilirubin from 5.0 to 9.9 mg/dL) IFALD incidence from 32% to 20%; in this population, infants with NEC showed the greatest improvement, with a reduction in IFALD from 67% to 42% [28]. Similarly, Wang et al. found that higher initial volumes (>15 mL/kg/day) and faster daily advancements (>10 mL/kg/day) resulted in a significantly lower incidence of IFALD in post-operative recovery of neonates with intestinal atresia, suggesting that higher-volume feeding may improve the prognosis of this specific group of surgical neonates [29].

#### 3.4.5. Feeding Advancement

The ESPGHAN Committee of Nutrition recommend that, once trophic feeding is tolerated (~20 mL/kg/day), the feeding volume should be gradually advanced to reach a caloric target of 115–140 kcal/kg/day in preterm neonates [72]. However, evidence on the optimal strategy for advancing EN are limited.

In neonates undergoing intestinal surgery, implementing standardized multidisciplinary protocols for postoperative EN, aiming at reducing practice variability, is associated with improved clinical outcomes, including faster achievement of nutritional goals and a reduced incidence and severity of IFALD [19,28,30,34,71,73,74]. Various refeeding protocols have been proposed in the literature [28,29,30,34,51,61,63,71,75]. Generally, feeding volume is increased by 10–20 mL/kg/day, with tolerance closely monitored through the assessment of abdominal distension, vomiting, stoma output, stool output, hydration status, and other clinical parameters [28,46,63,74,75]. Some experts recommend an upper acceptable limit of stool output of 40–50 mL/kg/day (ten bowel movements) [48,51,55], while others suggest a lower threshold of 20 mL/kg/day (six bowel movements) [63,76]. Whilst some of the patients can be easily managed adhering to a standard feeding protocol, in infants at high risk of IF, like patients with an ostomy, feeding advancement could be a real challenge. In these patients, the length and anatomy of the residual intestine and the stoma location must be taken into account; for example, infants with high jejunostomies may exhibit high stool output due to malabsorption, a condition that typically does not improve until intestinal continuity is restored [63,75]. Moreover, gastric output may not accurately reflect EN tolerance, as gastric hypersecretion is part of the intestinal adaptation process following bowel resection [77]. It has been proposed that in patients at high risk of IF, such as preterm infants with surgical NEC with or without an ostomy, modest enteral feeding could be started in the intermediate period, according to the patients’ general conditions, and a gradual increase in EN can be delivered during the recovery phase, when the inflammatory response has decreased or solved, with regard to the improvement in intestinal adaptation and/or ostomy output. On this issue, some authors suggest feeds should be temporarily withheld if ostomy output exceeds 3 mL/kg/h [63].

Based on the aforementioned considerations, an alternative approach has been used in some of the feeding guidelines published in the last years, which involves tailoring feeding advancement based on the risk of feeding intolerance, determined by patient’s weight and clinical course, residual bowel length, and surgical history (categorized as high, medium, or low risk) [34,75].

Overall, while it is essential to implement and adhere to standardized nutritional management protocols to optimize clinical outcomes, it is fundamental that these protocols must ultimately be tailored to meet the unique needs and characteristics of each patient.

#### 3.4.6. Role of Specific Nutrients

Current studies do not provide evidence-based data to establish clear recommendations regarding the appropriate single enteral nutrient intake for patients with IFALD.

In cases of IF, whether temporary (e.g., high jejunal stoma) or permanent (such as SBS), enteral lipid supplementation has been proposed based on several physiological principles. Lipids offer a higher caloric density per unit volume compared to other macronutrients and, being isosmotic, help prevent fluid loss, electrolyte imbalances, and dehydration. Animal studies suggest that, while a low-fat diet may compromise intestinal adaptation [78], a high-fat diet, particularly one enriched with long-chain polyunsaturated fatty acids (LCPUFA), enhances intestinal adaptation and absorption after bowel resection [79,80,81], although the underlying mechanisms remain partially unclear. Moreover, enteral lipid administration may stimulate bile flow via cholecystokinin-mediated gallbladder contraction [82]. Early enteral fat supplementation in infants with IF might also facilitate the reduction of parenteral lipid emulsions, especially soybean-based formulations, which are associated with an increased risk of liver injury and IFALD [83,84,85].

An RCT examined enteral fat supplementation in preterm neonates undergoing intestinal resection with a high (jejunal) or low (ileal) ostomy. In this study, patients received either standard enteral feeding or enteral feeding supplemented with safflower (omega-6) and fish oil (omega-3). Neonates in the lipid-supplementation group had lower conjugated bilirubin levels, required less intravenous lipids, and had a higher enteral caloric intake. However, the study has several limitations, including a small sample size, with only seven out of thirty-six patients meeting the criteria for SBS [33].

A case series of six infants with IF and prolonged PN showed improved cholestasis after temporarily removing PN-soybean emulsions, with or without enteral fish oil supplementation, suggesting that discontinuation of soybean emulsion alone may be beneficial in the treatment of IFALD [86]. However, another study described six PN dependent infants with SBS and IFALD who received enteral fish oil supplementation without changing the PN soybean emulsion. In this cohort, IFALD was completely reversed in four of the six infants within 5 ± 2.6 weeks (range 2–8 weeks) after starting enteral fish oil supplementation, proposing that enteral fish oil could be an effective adjunctive treatment for infants with IFALD who are able to tolerate some amount of EN due to sufficient small bowel length [34].

Overall, some experts recommend modulating fat intake and supplementing with enteral fish oil, tailoring the choice of lipid type to the patient’s clinical profile, including residual bowel length, surgical history, and risk of malabsorption [87]. Long-chain triglycerides (LCT) may slow intestinal transit and promote rehabilitation [82], whereas, in cases of LCT intolerance, MCT provide a more digestible and better-absorbed alternative, particularly when the colon is intact [88]. This integrated approach optimizes EN and reduces dependence on PN lipids, thereby enhancing overall nutritional status and potentially mitigating the risk and severity of IFALD.

## 4. The NOT IFALD Cholestatic Patient

### 4.1. Definition

Patients suffering from cholestasis not associated with IF can be affected by a wide range of diseases, as depicted in Figure 1. The common ground is the intestinal integrity in its length and function and the presence of a liver disorder resulting in cholestasis. The altered bile acid flow with severe depletion of bile acids in the intestinal lumen leads to fat malabsorption and the related complications of malnutrition and fat-soluble vitamin deficiencies. On the other hand, important differences can be identified among the underlying diseases affecting these patients. First of all, the duration of bile fluid impairment is not the same, from days to years, and in some cases, cholestasis can be reversible (for example bacterial sepsis). Moreover, the clinical status could be very different, with some patients having an acute disease (bacterial sepsis, galactosemia, hypopituitarism, or gallstones) and others suffering from a chronic liver disease (such as biliary atresia, α1-antitrypsin deficiency-A1AT, Alagille syndrome, progressive familial intrahepatic cholestasis-PFIC), possibly evolving in ESLD. In addition, we can distinguish genetic (A1AT, Alagille syndrome, PFIC, metabolic diseases) from acquired disorders (bacterial sepsis, PNAC). Furthermore, the time of presentation can vary, occurring within the first days of life (as in the case of galactosemia) or later on (as seen in biliary atresia), in both preterm or term infants. Finally, the outcome is highly variable, ranging from reversible and treatable diseases (such as galactosemia or sepsis) to more severe conditions that require surgery (like biliary atresia or choledochal cysts) or that evolve into ESLD (such as some genetic diseases or biliary atresia) and that need a hepatic transplantation during infancy.

### 4.2. Incidence

It is known that neonates are particularly prone to different forms of impairment in bile formation because metabolic demands might not be matched by adequate functional maturation in the first weeks of life [89]. However, estimating the global incidence of NOT IFALD cholestasis in newborns may be challenging and there are no clear data in the available literature. Scarce data are available in NICU patients, a few focusing exclusively on PNAC, sometimes without a clear distinction between IFALD and NOT IFALD patients [2,10]. Finally, NOT IFALD cholestasis in a NICU patient has often a multifactorial basis (prematurity, asphyxia, PN, chromosomal disorders, infections, sepsis, Rh alloimmunization), as shown in a retrospective study by Tufano et al.; in this paper, apparently not including IFALD patients, an overall incidence of neonatal cholestasis of 2% and an incidence of multifactorial cholestasis of 92% was described. Of note, in this cohort, 92% of the cholestatic patients were preterm infants [2]. In fact, in preterm infants, immaturity of the enterohepatic circulation, absence of feedings, intestinal inflammation or dysfunction, repeated bacterial or fungal infections, and use of PN may contribute to the occurrence of cholestasis, with an incidence of about 10–20% [90].

Among the different etiologies of neonatal NOT IFALD cholestasis, some data are available on the incidence of PNAC/PNALD. In a systematic review that tried to separate studies including exclusively IFALD or NOT IFALD patients, the incidence of PNAC/PNALD among NOT IFALD preterm and term infants/older children has been estimated at about 26% and 31% in the two age groups respectively [10]. This finding is in contrast with data from other authors that showed an inverse correlation between PNAC and birth weight [91]. Finally, the incidence of PNAC is directly proportional to the length of PN, with an incidence varying from 15.7% in patients receiving PN for 14–30 days up to 60.8% in patients receiving PN for >60 days [10].

### 4.3. Etiology

In the early 1970s, the differential diagnosis of the cholestatic newborn was limited. Biliary atresia accounted for approximately 25% of the cases; a small percentage of cases were ascribed to viral infections or were attributed to a handful of recognizable metabolic or inherited diseases (i.e., galactosemia, tyrosinemia, cystic fibrosis). The vast majority were designated with the default diagnosis of idiopathic neonatal hepatitis, because the underlying pathophysiology was unknown [92].

However, the diagnostic pathway for neonatal cholestasis has evolved in recent years, largely because of better understanding of biliary physiology and the concurrent expanding identification of new genetic causes of cholestasis with the advent of technological advances in gene sequencing, advanced microscopy and cell biology [93].

Nowadays, the largest diagnostic groups are biliary atresia (25–40%); α1-antitrypsin deficiency—A1AT (5–15%); other inherited forms of cholestasis, such as PFIC (10% to 20%); inborn errors of metabolism and congenital infections (including the TORCH infections), that cause respectively 20% and 5% of cases; hypopituitarism (5%); and PNAC, which is the commonest cause in preterm infants [94].

Different classifications of NOT IFALD etiologies of cholestasis have been proposed in the literature, the commonest considering the physiopathology of bile flow impairment, i.e., a defect of the intrahepatic production or the transmembrane transport of bile, or a mechanical obstruction of the biliary system, and identifying eight groups, according to the etiopathogenesis of hepatic dysfunction, as depicted in Figure 1.

### 4.4. The Role of Enteral Nutrition in the Prevention and Management of NOT IFALD Diseases

In addition to prompt recognition of diseases amenable to specific medical therapy (i.e.,: congenital toxoplasmosis, urinary tract infection, galactosemia, tyrosinemia, hypothyroidism) or early surgical intervention (biliary atresia, choledochal cyst), infants who suffer from cholestasis may benefit from optimization of nutrition to prevent complications.

Nutritional support in children with liver disease is usually dictated by the type of liver injury. Newborns affected by an inborn error of metabolism (i.e., galactosemia, tyrosinemia, glycogen storage, and Wilson disease) require specific dietary restrictions. Patients with fulminant hepatic failure may be well-nourished at presentation but will still require dietary interventions for management of acute complications such as hyperammonemia, fluid imbalance, and encephalopathy symptoms. Congenital or acquired infections may require some kind of nutritional support, depending on the duration of cholestasis and potential associated conditions (such as prematurity and PN need).

Among the several etiologies of the NOT IFALD cholestasis, biliary atresia and hepatic genetic and metabolic diseases are severe enough to determine long lasting malabsorption that justify a careful nutritional support. Optimizing EN in order to achieve a better nutritional status is of paramount importance in these diseases, as outcome of transplantation is related to nutritional status. In fact, supplemental enteral or parenteral feeding is the one intervention known to improve pre- and post-transplant growth and clinical outcomes in children with ESLD [95]. Under these premises, most of the available literature on nutritional support of NOT IFALD newborns is focused on patients with chronic liver diseases. In addition, some data are published about the role of EN in the prevention of PNAC/PNALD.

Therefore, in the following sections, we will focus on the abovementioned two subgroups of NOT IFALD cholestatic newborns, presenting data about EN in the prevention of PNAC/PNALD preterm infants and evidence for dietary management of patients with chronic liver diseases that may benefit from a long-lasting nutritional intervention.

### 4.5. The Role of Enteral Nutrition in the Prevention of PNAC/PNALD

Early enteral feeding is considered a key factor in preventing cholestasis in preterm infants [96]. By reducing exposure to PN, preserving intestinal integrity, supporting immune function, and minimizing the risk of bacterial translocation, enteral feeding contributes to the normalization of hepatobiliary function [97]. We have already mentioned two RCTs dating back from the 80s that showed decreased cholestasis with early enteral instead of intravenous protein administration in the very low birth weight infant [64,65]. These studies have also been identified as the only significant references about the effect of EN on prevention of PNAC/PNALD by a systematic review by Lauriti et al., where the authors found that although numerous studies have aimed to prevent PNAC, only a few have prospectively evaluated interventions [10]. In a recent case–control study in preterm infants born less than 32 weeks and diagnosed with PNAC, the administration of enteral omega-3 fatty acids was found to be associated with significantly shorter duration of cholestasis [98]. However, due to the retrospective nature of the study and the small sample size, more data are needed to prove the utility of enteral fish oil in preterm cholestatic patients.

Despite the lack of robust evidence, there is general agreement that the management of PNAC/PNALD in preterm infants should include the discontinuation of PN as soon as possible and the promotion of enteral feeding (including trophic feeding), which enhances bile flow, gallbladder contraction, and intestinal motility [51,60].

### 4.6. Enteral Nutrition Management of Chronic Cholestatic Liver Diseases

Moderate to severe malnutrition affects approximately 60% to 80% of infants with chronic liver diseases, increasing morbidity and mortality of patients [95]. Although the exact mechanisms remain unclear, multiple factors contribute to this condition. While initially malnutrition can be related to impaired digestion and nutrient absorption due to low intraluminal bile acid levels and increased energy demands, as the disease progresses, pathophysiology becomes more complex and, in addition, reduced oral intakes, abnormal nutrient metabolism, increased energy expenditure, recurrent infections, endocrine dysfunction, and enteropathy associated with cirrhosis and portal hypertension are key contributors [99]. Careful and personalized nutritional support should be started as soon as possible; this is of paramount importance, especially in newborns and infants, to optimize appropriate development and hepatic transplant results in patients at risk of ESLD.

Nutritional interventions in chronic liver diseases aim to face protein-calorie malnutrition, which is related to malabsorption of fat and a diversion of metabolism towards carbohydrates and protein utilization as sources of energy [100]; the latter process ultimately leads to depletion of glycogen stores and fat-free mass from liver and muscles.

The recommendations we present in the following sections are included in a recent joint position paper by the North American Society for Pediatric Gastroenterology, Hepatology, and Nutrition and the European Society for Pediatric Gastroenterology, Hepatology, and Nutrition published in 2019 [99]. Lacking robust evidence, most of the recommendations consist of expert opinions or are dictated by clinical practice; some of them are essentially based on metabolic and pathophysiologic studies in animals or small groups of patients that date back to the end of the last century. A summary of the recommendations is shown in Table 3.

#### 4.6.1. Energy Requirements

Establishing energy needs for patients with chronic cholestatic liver diseases can be challenging in the clinical setting. Energy requirements depend on resting energy expenditure (REE), level of physical activity, and severity of maldigestion/malabsorption. In particular, commonly used equations for REE, such as the Schofield equations (based on weight, or weight and height), are often inaccurate for these patients; some authors reported increased energy expenditure from a hypermetabolic state probably due to the intracellular activation of thyroid hormone by bile acids [102]. Moreover, energy requirements can be different according to disease severity. In the late 1980s the first evidence of protein-energy malnutrition in severe liver disease was published, estimating a 29% higher calorie need in patients affected by biliary atresia and cirrhosis in comparison to healthy controls [100]. Finally, energy demands can be further increased in case of complications, such as episodes of sepsis from peritonitis, cholangitis, or variceal bleeding [101].

Based on this evidence, it is generally recommended that infants with chronic cholestatic liver disease can require 125 to 140% of the recommended caloric requirement based on ideal body weight. For the smallest infants, this may mean a daily caloric goal of 150 to 160 kcal/kg per day. Indirect calorimetry for a more accurate estimation of energy needs should be used when available to guide energy provision, especially in malnourished patients [103].

#### 4.6.2. Fluids and Electrolytes

The intake of water and electrolytes should be normal for the infant and child’s weight, unless ascites is present, indicating the need for restriction. A minimum intake of sodium of 1 mmol/kg/day and potassium of approximately 2 mmol/kg/day is generally adequate [99].

In preterm infants Ca and P absorption rates range between 30–70% and 70–90%, respectively. Of note, unabsorbed free fatty acids in patients with cholestasis can bind to dietary calcium, leading to gastrointestinal calcium additional losses, thus contributing to metabolic bone disease. Therefore, if a Ca intake of 3.0–5.0 mmol (120–200 mg/kg/d) and a P intake of 2.2–3.7 mmol (70–115 mg/kg/d) are recommended in healthy preterm infants, higher supplementations can be needed in cholestatic patients.

#### 4.6.3. Carbohydrates

Carbohydrate needs are difficult to define in patients affected by chronic cholestatic liver diseases. Nonetheless, recent guidelines report that carbohydrate requirements in newborns and infants with cholestasis are typically 40–60% of their total calories and depend on associated comorbidities [99]. Carbohydrates represent a major source of energy, and can be particularly useful for increasing caloric intake. To meet energy and glucose needs, short-chain polymers, such as maltodextrins, are commonly used, because their low osmotic load helps prevent diarrhea.

Carbohydrate needs may be variable. On the one hand, patients may suffer from hypoglycemia, due to depletion of glycogen stores (which are particularly low in preterm infants), hepatic metabolic dysfunction, and hampered gluconeogenesis that are associated with disease progression. On the other hand, hyperglycemia is also a possible concern, due to potential insulin resistance that could be exacerbated in stressed neonates.

#### 4.6.4. Proteins

It is well-known that an adequate amino acid intake is crucial for protein accretion in neonates and infants, and a minimum intake of 1.5 to 2.0 g/kg per day is required to avoid catabolism and allow for growth [72]. In patients with chronic and ESLD, protein requirements are typically increased, due to protein loss, increased amino acid oxidation, reduced hepatic production and poor nutritional status. In children with biliary atresia and cirrhosis, increased protein oxidation in spite of a 2 g/kg/d protein intake resulted in a virtually zero nitrogen balance and even in oxidation of endogenous proteins [100]. Therefore, to sustain growth, a protein/energy ratio of 10% and an intake of around 2–3 g/kg/day is probably needed to maintain a positive nitrogen balance [101]. In the absence of encephalopathy, hyperammonemia up to 120 mmol/L is well tolerated in newborns and does not require protein restriction. Restriction is rarely necessary unless related to a urea cycle disorder or encephalopathy due to liver failure and portosystemic shunts. In such cases, intake should be limited to 0.5–1.0 g/kg/day, though long-term restriction below 2 g/kg/day should be avoided to prevent muscle protein catabolism [100].

Additionally, it has been described an increase in aromatic amino acids (AAAs) and decrease in branched-chain amino acids (BCAAs) due to the abnormal protein use by the liver in chronic liver diseases [104]. Studies in animals and in children with ESLD have demonstrated some beneficial effects of a formula enriched with BCAAs [105,106]; however, their use still remains debated.

#### 4.6.5. Lipids

Lipids are an essential component of an infant’s diet as they support proper growth and neurological development while providing essential long-chain polyunsaturated fatty acids (LCPUFAs) and fat-soluble vitamins. The lipid requirements of cholestatic infants depend on their nutritional status, the extent of gastrointestinal losses, and the severity of liver dysfunction, typically accounting for 30–50% of total caloric intake [99,101]. Despite the possible development of steatorrhea, animal studies suggest that overall increasing the dietary fat intake helped to maintain net fat uptake compared with control conditions [107,108].

Medium-chain triglycerides (MCTs) remain a key component of supplementation for cholestatic patients. MCTs contain fewer kilocalories per gram than LCTs (8.3 kcal/g MCT vs. 9 kcal/g LCT) and are not a source of essential fatty acids. However, unlike LCTs, which require bile salts for digestion and absorption, MCTs can be passively diffused through enterocytes directly into the portal circulation [108]. Therefore, supplementation with MCT oils or MCT-containing formulas are recommended for infants with cholestasis [95]. Limited evidence suggests that the optimal MCT/LCT ratio for supplementation should be of 30% to 70% (MCT/LCT mix), but there is still no consensus on the optimal MCT/LCT ratio supplementation for cholestatic patients [99,101]. Finally, MCTs intake exceeding 80% of total fat energy should be avoided to prevent essential fatty acid (EFA) deficiencies [99]. EFAs, such as linoleic acid and α-linolenic acid, must be included in the diet of cholestatic newborns, especially preterm infants, as they serve as precursors for LCPUFAs, including arachidonic acid and docosahexaenoic acid, which are crucial for brain and retinal neurodevelopment [109]. While in healthy subjects, in order to prevent EFA deficiency, an amount of LCT of about 3% of total fat calories is required, in the context of cholestasis, patients may require much higher intakes, up to 10% of total energy intake [101].

#### 4.6.6. Fat Soluble Vitamins

As already mentioned, fat soluble vitamins are a lipid component of the diet whose absorption can be compromised if the bile acid level in the intestinal lumen is below a critical micellar concentration. This is more frequent when direct bilirubin serum levels are greater than 2.0 mg/dL [112].

Some data on the incidence of fat-soluble vitamin deficiencies in infants and children affected by chronic liver diseases are available in the current literature [12], but we did not find studies exploring this issue in cholestatic preterm or ex-preterm infants.

When cholestasis begins, vitamin storages present at birth can be rapidly depleted, especially in preterm infants; hence, in the cholestatic newborn, the first biochemical and clinical signs of fat-soluble vitamin deficiency may appear in the first months after birth. Therefore, vitamin supplementation should be started from the earliest stage of neonatal cholestasis, and infants should be screened regularly for monitoring their vitamin status, adjusting dosages to the specific needs and avoiding the onset of side effects. As vitamin needs can be extremely variable, depending on the severity of cholestasis and the intestinal absorbative capacity, often reduced in preterm infants, separate supplementation of the different vitamins is the best strategy to individualize therapies. Moreover, in the newborn period, especially in preterm infants, some important considerations about vitamin status and metabolism have to be made. In fact, preterm infants born before 32 weeks often have low vitamin A levels and reduced retinol-binding protein, their primary carrier, and may require higher supplementation than term infants. Moreover, high dose intramuscular vitamin A for prevention of bronchopulmonary dysplasia is still not always prescribed in NICUs. As far as Vitamin D is concerned, even before 28 weeks of gestational age, its absorptive and metabolic pathways are fully operative. However, suboptimal intakes of calcium, phosphorus and vitamin D often lead to metabolic bone disease in these patients. Despite lack of robust evidence, a vitamin D supplementation ranging from 300 to 1000 UI/die, according to birth weight, has been proposed in the last position paper from ESPGHAN committee in 2022 for the “healthy” preterm infant [72], and higher doses could be necessary for cholestatic patients. This is true also for vitamin E supplementation, whose recommended daily intake for a preterm infant has been estimated as 2.2–11 mg/kg/d, and vitamin K, despite routine prophylactic intramuscular supplementation at birth and additional intakes from PN, infant milk formula, and human breast milk fortifiers [72]. Nonetheless, in the literature, studies on fat soluble vitamin supplementation in preterm and ex-preterm cholestatic infants are lacking. Moreover, the available indications for supplementation in children have not been standardized and different routes of administration and wide dosage ranges have been published. A scoping review on this topic has recently been published by Degrassi et al. [12], comparing all the suggestions found in the available literature about fat soluble vitamins supplementation in cholestatic patients. Here, we report the recommendations of the Joint Position Paper of the North American Society for Pediatric Gastroenterology, Hepatology, and Nutrition and the European Society for Pediatric Gastroenterology, Hepatology, and Nutrition published in 2019 on supplementation and monitoring in cholestatic patients affected by chronic liver diseases, as depicted in Table 2. These recommendations are based mostly on old studies conducted in small groups of infants and children with chronic liver diseases [12], and we could not find more recent evidence to further define what is the best supplementation to use.

Finally, some data are available about the use of aqueous preparations of fat-soluble vitamins, that can be transported directly into the portal circulation without the need for bile salts. A study conducted in infants with biliary atresia showed the efficacy in reducing fat soluble vitamin deficiencies of a water-soluble ADEK multivitamin formulations [110]; however, separate supplementation is often needed in these patients. D-Alpha-tocopheryl polyethylene glycol 1000 succinate is a water soluble source of vitamin E that has been demonstrated having a better bioavailability in comparison to the previous formulations used in children with cholestasis; after its approval in Europe, a multicenter prospective study, including cholestatic patients from 0 to 232 months, demonstrated safety and efficacy in restoring and/or maintaining sufficient serum vitamin E level in the majority of the study patients, avoiding the need for intramuscular vitamin E injections [111].

#### 4.6.7. Water Soluble Vitamins and Trace Elements

Even if not unanimously reported in the available literature, some authors advocate the need to supplement also water-soluble vitamins in cholestatic patients. Multivitamin formulations can be used, and twice the RDA is the recommended dose, regardless of whether the patient’s vitamin status is difficult to evaluate [101,113]. Malabsorption can involve also some trace elements, such as zinc, selenium, or iron, and they should be supplemented according to the plasma levels [101].

#### 4.6.8. Human Milk Versus Infant Milk Formulas

Given the lack of RCTs, the nutritional approach to a patient affected by a cholestatic chronic liver disease is mostly expert-opinion based.

Overall, the dietary management of a patient with a cholestatic liver dysfunction can be tailored according to the three stages of the disease: early, chronic, and end-stage. In the early stage of disease, when the degree of cholestasis is still milder, human breast milk or standard infant milk formulas may be adequately absorbed. In preterm infants, human breast milk can be supplemented with breast milk fortifiers. If breastfeeding is not possible, an MCT-enriched formula should be used. More severe cholestasis will require MCT oil supplements or infant milk formulas containing larger amounts of MCTs, adequate MCT/LCT ratio, and sufficient amounts of EFA. Sometimes, to meet the energy needs of patients, increasing the caloric density of the formula to 0.8–1 kcal/mL can be an option to consider, using supplements or choosing concentrated formulas containing MCTs and maltodextrins.

There are still very few data about BCAAs-enriched formulas to recommend its use. However, an infant milk formula for the dietary management of acute and chronic liver disease is commercially available; it is composed of dried glucose syrup, 49% MCT, 30% of total protein as BCAAs and low sodium content, but there are not published studies on this product, other than one report on its safety and tolerability, without evidence of beneficial effects on patients’ growth [101].

#### 4.6.9. Mode of Delivery

As previously described for IFALD patients, oral nutrition should always be encouraged whenever possible, if sufficient energy and nutrient supply can be secured. Small and frequent feeding may be useful to this purpose and to tackle early satiety, prevent hypoglycemia, and avoid muscle catabolism.

However, to meet high energy needs and especially in end-stage cholestasis, when patients can suffer from anorexia, enteral feeding with a nasogastric tube may be the best option. Bolus feeding is considered more physiological and is the first choice in most cases. However, a study in infants affected by biliary atresia and ESLD, comparing an MCT-fortified formula administered orally vs. 18 h enteral administration via nasogastric tube, showed that EN prevented malnutrition and growth impairment [114].

To preserve oral nutrition, another option to evaluate is exclusively night-time nasogastric tube feeding.

Jejunal tube feeding or an endoscopic gastrostomy (if portal hypertension is mild) are to be considered only if nasogastric tube feeding is not feasible and if a multidisciplinary team can provide active follow-up and care for the child [101].

## 5. The Effect of Nutritional Management on the Growth of the Cholestatic Newborn

### 5.1. IFALD Cholestatic Patients

Growth failure and altered body composition have been described in follow-up studies of infants with SBS and IFALD patients [115,116,117,118,119,120,121]. This specific population struggles to maintain adequate growth, secondary to malabsorption and the subsequent difficulty to meet the high nutritional needs required during this time of rapid growth and brain development.

Evidence on the effect of intestinal rehabilitation programs on growth of IFALD patients is lacking. Actually, during the first weeks/months, patients rely often on PN for their growth [122], and the decision to advance EN and wean PN is based on whether the patient is growing appropriately, and the severity of any PN associated complications. However, some data exist on the effect of a few EN strategies on growth of IF patients, some of them suffering from liver disease (Table 4).

A small RCT cross-over study including ten patients with SBS (two cholestatic patients) failed to show a difference in weight gain between hydrolyzed and non-hydrolyzed formula over a 60-day period [24].

A retrospective single center study of 163 neonates including IFALD patients showed that older age at onset of enteral feeds independently predicted lower discharge weight; peak conjugated bilirubin was not associated in multivariate analysis with 6-month weight percentile [123].

In a retrospective review of medical charts of 10 neonates with enterostomies and poor growth, Malcolm et al. reported significant short-term improvements in both ostomy output and weight gain after introducing enteral LCT supplementation [124]. However, an RCT evaluating the effect of enteral LCT supplement added to an omega-6 based fat, to maintain appropriate fat ratios in preterm infants with an enterostomy (cholestatic patients included), found greater weight and length gain only after re-anastomosis in the treatment group [33].

Animal studies have demonstrated that increased enteral fat concentration prevented postoperative catabolic responses and increased lean mass after small bowel resection, while low fat diets, despite comparable caloric intake, negatively impact adaptation, as evidenced by decrease in body weight [78,125]; however, no data on SBS and IFALD patients are available on this topic.

### 5.2. NOT IFALD Cholestatic Patients

The PN management of a preterm patient is one of the main determinants of the development of PNAC/PNALD [10]. Some authors suggest that following standardized enteral feeding guidelines trying to reduce the length of PN use can result in better growth and reduced morbidity in very-low birth weight infants, including PNAC/PNALD [126,127,128]. Our research retrieved only one paper reporting the impact of specific EN strategies on growth of PNAC/PNALD patients. In this single center retrospective case–control study, 24 preterm infants born less than 32 weeks of gestation affected by PNAC (direct bilirubin > 2 mg/dL) and who received enteral omega-3 fatty acids supplementation (1 g/Kg/d) were compared with gestational age, gender, and direct bilirubin level matched controls who did not receive enteral omega-3 fatty acids supplementation. Infants who received enteral omega-3 fatty acids had significantly shorter duration of cholestasis (*p* = 0.025) and a higher average daily weight gain (about 3 g/day) (*p* = 0.011) than their controls [98].

There are very few RCT trying to elucidate the effect of specific nutritional approaches for patients with chronic cholestatic liver disease to achieve better growth outcomes (Table 4). As already mentioned, in an RCT study including patients affected by biliary atresia and ESLD, 15 infants were randomized to receive an MCT-fortified formula administered orally vs. 18 h enteral administration via nasogastric tube; this study showed that EN prevented malnutrition and growth impairment [114].

Another cross-over study randomized 19 children with ESLD fed by nasogastric infusion to receive for 8 weeks two matched isocaloric and isonitrogenous nutritional formulations differing only in their BCAAs content. During BCAAs supplementation, improved weight and height and significant increase in mid-upper arm circumference and subscapular skinfold thickness were detected [105].

A recent review on the impact of MCTs on fat absorption, growth, and nutritional status of children affected by chronic liver diseases found that MCT supplementation was associated with greater fat absorption and improved growth in some children, without any effect on growth by the use of higher MCTs percentages in the diet [108].

## 6. Strengths and Limitations of This Study

This is a comprehensive review exclusively focused on the role of enteral nutrition in cholestasis and its management in the vulnerable cohort of newborn patients. We presented data published over a wide period of time in which neonatal research has progressively increased and we tried to summarize available evidence on this topic.

**Figure 2:**
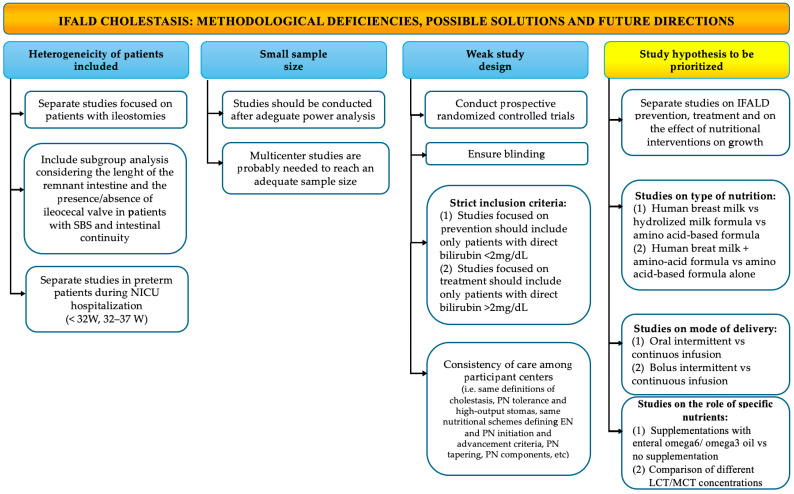
Methodological deficiencies, possible solutions, and future research directions in IFALD neonatal cholestasis. IFALD: intestinal failure associated liver disease; LCT: long-chain triglycerides; MCT: medium-chain triglycerides; NICU: neonatal intensive care unit; SBS: short bowel syndrome; W: week.

**Figure 3:**
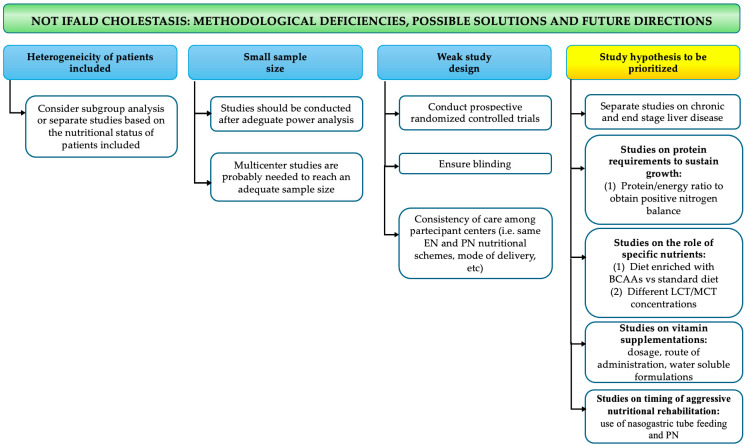
Methodological deficiencies, possible solutions, and future research directions in NOT IFALD neonatal cholestasis. BCAAs: branched-chain amino acids; EN: enteral nutrition; IFALD: intestinal failure associated liver disease; LCT: long-chain triglycerides; MCT: medium-chain triglycerides; PN: parenteral nutrition.

However, some limitations to this review need to be mentioned. First, the quality of the studies varied considerably; most of the studies were small and retrospective, and the statistical analysis not always included a multivariate or regression analysis. Studies were heterogeneous in patient selection (definition of the IFALD or NOT IFALD population), and outcome measures, especially in the timing of growth assessment. All these limitations have been addressed in Figure 2 and Figure 3, where methodological deficiencies in the available literature are highlighted and possible solutions and future directions are suggested.

## 7. Conclusions

EN undoubtedly play a pivotal role in the clinical management of patients with a cholestatic disorder, both in the contest of IF or of liver dysfunction without intestinal impairment. The early initiation of an appropriate nutritional program is of paramount importance, starting from the neonatal period onward. However, despite the general agreement of experts and societies on many facets of the management, available data, published over a period of more than 50 years, are often retrospective in nature, and robust evidence is still lacking for many aspects of EN strategies to be used. Lack of evidence is even more striking in the fragile population of preterm infants, who may suffer from cholestasis and chronic liver diseases, where data on their specific management and, above all, on their long-term outcomes, in terms of growth and neurodevelopment, are still very scarce. Therefore, in the near future, new studies, especially multicenter RCTs, are needed to guide neonatologists’ decisions and optimize care of cholestatic newborn patients.

## Figures and Tables

**Table 1:** Main etiologies of neonatal cholestasis in patients with intestinal failure-associated liver disease (IFALD) and in those suffering from liver dysfunction without intestinal impairment (NOT IFALD).

	Category	Main Diseases
IFALD	Intestinal failure	Necrotizing enterocolitis, intestinal obstruction, congenital intestinal malformations, volvulus, short bowel syndrome, intestinal neuromuscular motility disorders, congenital enteropathies
NOT IFALD	Obstructive anomalies of biliary system	Biliary atresia, choledochal cysts, cholelithiasis, thick bile syndrome, spontaneous perforation of common bile duct
	Infections	Viral: CMV, rubella, HSV 1,2,6, parvovirus B19, hepatitis A, B, C, chickenpox, adenovirus, enterovirus, coxsackievirus Bacterial: Syphilis, Listeria, congenital TBC, sepsis, urinary tract infections Parasitic: Toxoplasma
	Toxic	Parenteral nutrition-associated cholestasis (PNALD), Drugs (Ceftriaxone, Erythromycin, Rifampicin)
	Endocrine	Hypothyroidism, panhypopituitarism, adrenal insufficiency
	Immune	Neonatal hemochromatosis (gestational alloimmune liver disease), hemophagocytic lymphohistiocytosis, congenital systemic lupus erythematosus
	Cardiovascular (hypoxia, ischemia, hepatic congestion)	Perinatal asphyxia, in utero growth restriction, cardiovascular diseases
	Genetic and metabolic disorders	Cystic fibrosis, alpha 1 antitrypsin deficiency, Alagille syndrome, galactosemia, tyrosinemia type I, hereditary fructose intolerance, bile acid synthesis defects, congenital hepatic fibrosis, citrin deficiency, bile acid conjugation defects, fatty acid oxidation defects, glycogen storage disease type IV, mitochondrial respiratory chain disorders, Niemann–Pick type C disease, peroxisomal disorders, progressive familial intrahepatic cholestasis, bile transport defects, cytoskeleton defects, Smith–Lemli–Opitz syndrome, Down syndrome
	Other	Idiopathic neonatal hepatitis (transient neonatal cholestasis), malignancy

**Table 2:** Studies on enteral feeding strategies in newborns and infants with intestinal injury and their impact on cholestasis.

	Reference	Study Design	Study Period	Sample	Main Inquiry	Effects on Cholestasis
Type of nutrition	Ksiazyk (2002) [24]	Randomized, cross-over, double blind	NS	10 patients with SBS (aged 6 weeks–8 months) 3 out of 10 with cholestasis	Hydrolyzed protein vs. standard formula on growth and development of children with SBS.	Not mentioned. Notes: no absorptive advantage, difference in energy expenditure, or weight gain in administering hydrolyzed vs. non-hydrolyzed proteins.
	Kulkarni (2013) [25]	Retrospective cohort	2010–2011	67 newborns receiving PN for >4 weeks	Human breast milk vs. milk formula in preventing PNALD	Lower maximum DB plasma concentration and lower prevalence of PNALD in human breast milk-fed infants compared with formula milk group (35 vs. 73%; *p* = 0.008).
	Andorsky (2001) [26]	Retrospective cohort	1986–1998	30 neonates with SBS and dependence on PN > 90 days after surgery	Risk factors for duration of PN and peak serum DB concentration	EN with human breast milk or an amino acid-based formula was associated with a shorter duration of PN; EN with protein hydrolysate formula was associated with a lower peak DB concentration. Not confirmed in multivariate analysis.
Mode of delivery						Not found
EN initiation and advancement	Ekingen (2005) [27]	RCT	2000–2003	56 newborns who underwent upper abdominal surgery	Compare early enteral feeding (12 h post-surgery) vs. feeding initiation after ileus resolution	Not significant. Notes: Early EN (3 to 5 mL of human breast milk every hour through NGT) initiated at a mean of 12 h post-surgery promoted earlier stool passage, shorter nasogastric feeding duration, and faster full oral feeding.
	Shakeel (2020) [28]	Multicenter, Prospective with historical controls	2007–2018	409 infants < 6 months of age at risk of IF after surgery requiring >7 days of PN	Incidence of IFALD and time to reach 50% of target EN calories before and after implementation of feeding strategies	Higher initial volumes of minimal EN up to 20 mL/kg/day, and faster daily feeding advancements by 20 mL/kg/day reduced the incidence of moderate IFALD from 32% to 20%.
	Shores (2018) [19]	Prospective with historical controls	2007–2016	164 infants < 6 months of age at risk of IF after surgery requiring >7 days of PN	Incidence of IFALD and time to reach 50% of target EN calories before and after implementation of EN strategies	Incidence of IFALD decreased from 71 to 51% (*p* = 0.031), and median peak DB decreased from 5.7 to 2.4 mg/dL (*p* = 0.001).
	Wang (2022) [29]	Retrospective cohort	2019–2021	32 neonates requiring PN after surgery for intestinal atresia	High-dose vs. low-dose feeding strategy	Higher initial volumes (>15 mL/kg/day) and faster daily advancements (>10 mL/kg/day) were associated with significantly lower incidence of IFALD.
	Savoie (2016) [30]	Retrospective cohort	2007–2011	163 infants who underwent intestinal surgery	Time to reach full EN in neonates fed without standardized feeding regimen vs. standardized strategy based on body weight and percentage of remaining small bowel	Cholestasis was less severe in the post-implementation group of infants and human breast milk use increased.
	Tillman (2014) [31]	Retrospective cohort	2007–2011	64 newborns with surgical NEC	Incidence of IFALD before and after implementation of feeding strategies	Incidence of IFALD improved from 73% before to 42% after guideline implementation (*p* = 0.01) and degree of hyperbilirubinemia was less severe.
	Garg (2023) [32]	Retrospective cohort	2013–2018	91 preterm infants with surgical NEC, 62/91 developed cholestasis	Clinical factors and outcomes of cholestasis in preterm infants with surgical NEC	Time from surgery to EN initiation and the duration of postoperative ileus were independently associated with mild to moderate cholestasis at two months of age.
Role of specific nutrients	Yang (2014) [33]	RCT	NS	37 preterm infants < 2 months of age with jejunostomy or ileostomy (7/37 with SBS)	Effects of early enteral fish oil supplementation on duration of PN before bowel re-anastomosis	Neonates receiving EN supplemented with safflower (omega-6) and fish oil (omega-3) had lower DB levels, required less intravenous lipids, and achieved higher enteral intake compared to those on standard EN.
	Tillman (2011) [34]	Retrospective case series	NS	6 PN-dependent infants with SBS and IFALD	Enteral fish oil for treatment of IFALD	IFALD reversed in 4 of 6 infants within 5 ± 2.6 weeks (range 2–8 weeks) after starting enteral fish oil supplementation.

Abbreviations: EN: enteral nutrition; PN: parenteral nutrition; DB: direct bilirubin; PNALD: parenteral nutrition-associated liver disease; IFALD: intestinal failure-associated liver disease; NEC: necrotizing enterocolitis; SBS: short bowel syndrome; NS: not specified; NGT: nasogastric tube.

**Table 3:** Recommendations for enteral nutrition support in children with NOT IFALD cholestasis.

Energy Nutrient	Requirement		Comments	Reference
Energy	125–140% of the recommended caloric requirement based on ideal body weight; smallest infants may require 150–160 kcal/kg/day		The Schofield equations for REE are often inaccurate; complications like sepsis, cholangitis, or variceal bleeding can further raise demands.	[100,101,102,103]
Fluids and Electrolytes	Normal fluid intake Sodium: 1–2 mmol/kg/day Potassium: ~2 mmol/kg/day		In case of ascites, fluid restriction may be required. Calcium and phosphorus needs may be higher due to fat malabsorption (minimum intakes in preterms are Ca: 3.0–5.0 mmol/kg/day; P: 2.2–3.7 mmol/kg/day).	[99]
Carbohydrates	40–60% of total calories		Maltodextrins preferred due to low osmotic load. Hypoglycemia and hyperglycemia may both occur.	[99]
Proteins	2–3 g/kg/day (higher needs in preterm infants); restriction to 0.5–1.0 g/kg/day only in case of encephalopathy		Higher needs due to increased oxidation and protein loss (up to 130–150% of requirements for age); BCAA-enriched formulas may be beneficial but evidence is limited.	[100,101,104,105,106]
Lipids	30–50% of total caloric intake		Start: MCTs/LCTs = 30/70% of total fat calories; MCTs from 30% up to 70% are recommended due to better absorption, in case of poor growth; MCTs intake >80% should be avoided; LCTs required to prevent EFA deficiency (minimum 3% of total fat calories, up to 10% in cholestasis).	[95,99,101,107,108,109]
Fat-Soluble Vitamins	Vitamin A	Oral <10 kg–5000 IU/day >10 kg–10,000 IU/day IM: 5000–10,000 UI/die OR up to 50,000 UI/month OS 2000–5000 IU/day	Start supplementation early, monitor regularly. Separate supplementation of the different vitamins is the best strategy to individualize therapies. Supplementation with all fat-soluble vitamins together may improve their absorption. Higher supplementation of vitamins may be required in cholestatic preterm infants (<32 weeks).	[12,72,110,111,112]
	Vitamin D (Cholecalciferol)	OS 15–25 IU/kg/day		
	Vitamin E (TPGS)	OS 2–5 mg/day		
	Vitamin K	IM <5 kg: 1 mg/kg every 2 weeks; >5 kg: 10 mg every 2 weeks		
Water-Soluble Vitamins and Trace Elements	Twice the RDA is the recommended dose (regardless of the patient’s vitamin status that is difficult to evaluate)		Multivitamin formulations can be used; zinc, selenium, and iron should be supplemented according to plasma levels.	[101]
Human milk and infant milk formulas	MCT-enriched formulas if breastfeeding is not possible; consider increasing caloric density of formula to 0.8–1 kcal/mL using supplements or choosing concentrated formulas containing MCTs and maltodextrins		BCAA-enriched formulas available, but evidence for benefits is limited.	[101,113]

REE: resting energy expenditure; BCAA: branched-chain amino acid; MCTs: medium-chain triglycerides; LCTs: long-chain triglycerides; EFA: essentially fatty acids; TPGS: D-alpha-tocopheryl polyethylene glycol 1000 succinate; RDA: Recommended Dietary Allowance; OS: oral somministration; IM: intramuscular injection.

**Table 4:** Studies on enteral feeding strategies in cholestatic newborns and infants with or without intestinal injury and their impact on growth.

	Reference	Study Design	Study Period	Sample	Main Inquiry	Effects on Growth
IFALD cholestasis	Ksiazyk (2002) [24]	Randomized, cross-over, double blind	NS	10 patients with SBS (aged 6 weeks–8 months) 3 out of 10 with cholestasis	Hydrolyzed protein vs. standard formula on growth and development of children with SBS	No absorptive advantage, difference in energy expenditure or weight gain in administering hydrolyzed vs. non-hydrolyzed proteins
	Niccum (2019) [123]	Retrospective cohort	2014–2017	163 newborns receiving PN for >5 days	Association between cholestasis (DB > 2 mg/dL) and weight percentiles at hospital discharge and 6 months of age	Weight percentiles in cholestatic infants were lower both at hospital discharge (14 ± 19 vs. 24 ± 22, *p*-value < 0.005) and at 6 months of age (24 ± 28 vs. 36 ± 31, *p*-value = 0.05). Peak conjugated bilirubin was not associated in multivariate analysis with 6-month weight percentile
	Yang (2014) [33]	RCT	NS	37 preterm infants < 2 months of age with jejunostomy or ileostomy (7/37 with SBS)	Effects of early enteral fish oil supplementation on weight gain before bowel re-anastomosis (secondary outcome)	Neonates receiving EN supplemented with safflower (omega-6) and fish oil (omega-3) had greater weight and length gain only after re-anastomosis (weight: 20 ± 9 vs. 27 ± 11, *p* < 0.05 and length: 0.9 ± 1.3 vs. 2.1 ± 1.5, *p* < 0.05 in controls and treatment group, respectively)
NOT IFALD cholestasis	Thavamani (2018) [98]	Retrospective case–control study	2011–2017	48 preterm infants born less than 32 weeks who developed PNALD	Effect of enteral omega-3 fatty acids supplementation (1 g/Kg/d) on postnatal growth	Infants who received enteral omega-3 fatty acids supplementation had higher average daily weight gain than their controls (22 ± 3 vs. 19 ± 4 g/day, *p* = 0.011)
	Macías-Rosales (2016) [114]	RCT	2009–2011	15 infants with a diagnosis of biliary atresia waiting for liver transplantation	Effect of oral vs. 18 h enteral administration via NGT of an MCT-fortified formula for 12 weeks on growth	Length/age and head circumference dropped in the per os group while it remained stable in the enteral nutrition group
	Chin (1992) [105]	Randomized cross-over study	1989–1990	19 infants and children with ESLD waiting for liver transplantation	Effect of two matched isocaloric and isonitrogenous nutritional formulations differing only in their BCAAs content fed by nasogastric infusion over a period of 8 weeks on growth	During BCAAs supplementation, improved weight (difference: 0.41 ± 0.16, *p* < 0.05) and height (difference: 0.50 ± 0.21, *p* < 0.05), and significant increase in mid-upper arm circumference (*p* < 0.05) and subscapular skinfold thickness (*p* < 0.02) were detected

Abbreviations: EN: enteral nutrition; ESLD: end stage liver disease; PN: parenteral nutrition; DB: direct bilirubin; PNALD: parenteral nutrition-associated liver disease; SBS: short bowel syndrome; NGT: nasogastric tube.

## Abbreviations

The following abbreviations are used in this manuscript:

NICU	Neonatal intensive care unit
AAP	American Academy of Pediatrics
IF	Intestinal failure
NEC	Necrotizing enterocolitis
SBS	Short bowel syndrome
IFALD	Intestinal failure associated liver disease
PN	Parenteral nutrition
EN	Enteral nutrition
PNAC	Parenteral nutrition-associated cholestasis
PNALD	Parenteral nutrition-associated liver disease
ESLD	End-stage liver disease
RCT	Randomized clinical trial
VEGF	Vascular endothelial growth factor
EGF	Epidermal growth factor
SIBO	Small intestinal bacterial overgrowth
LCPUFA	Long-chain polyunsaturated fatty acids
PFIC	Progressive familial intrahepatic cholestasis
TORCH	Toxoplasmosis, Others, Rubeola, Cytomegalovirus, Herpes
AAAs	Aromatic amino acids
BCAAs	Branched-chain amino acids
MCT	Medium-chain triglycerides
LCT	Long-chain triglycerides
EFA	Essential fatty acid
RDA	Recommended Dietary Allowances
REE	Resting energy expenditure
